# Emotional Response and Changes in Heart Rate Variability Following Art-Making With Three Different Art Materials

**DOI:** 10.3389/fpsyg.2018.00968

**Published:** 2018-06-18

**Authors:** Shai Haiblum-Itskovitch, Johanna Czamanski-Cohen, Giora Galili

**Affiliations:** ^1^Emili Sagol Creative Arts Therapies Research Center, School of Creative Arts Therapies, Faculty of Social Welfare and Health Sciences, University of Haifa, Haifa, Israel; ^2^Department of Psychiatry, Faculty of Medicine, The University of Arizona, Tucson, AZ, United States; ^3^The Department of Education and Psychology, The Open University of Israel, Ra’anana, Israel

**Keywords:** art therapy, heart rate variability, emotional response, expressive therapies continuum, art-making

## Abstract

Art therapy encourages the use of art materials to express feelings and thoughts in a supportive environment. Art materials differ in fluidity and are postulated to thus differentially enhance emotional response (the more fluid the material the more emotion elicited). Yet, to the best of our knowledge, this assumption has not been empirically tested. The current study aimed to examine the emotional and physiological responses to art-making with different art materials. We were particularly interested in vagal activity, indexed by heart rate variability (HRV), because of its association with numerous health related outcomes. In this study, 50 adults (mean age 33 ± 10.27 years, 52% males) participated in a repeated measures experiment, in which they were requested to draw with three art materials (order randomized) differing in their level of fluidity (pencil, oil-pastels, and gouache paint) intermittent with periods of music. We measured the emotional response to art-making with each material using a self-report measure and matrices of HRV using a wearable electrocardiogram device. We calculated two indices of HRV, one indicative of parasympathetic nervous system (PNS) activity, and one indicative of sympathetic nervous system (SNS) activity. Art-making with gouache paint and oil-pastels resulted in improved positive mood, while pencil did not. Art-making explained approximately 35% of the variability in parasympathetic reactivity, which may indicate changes in emotional regulation processes during the art-making task. Yet, fluidity was not sufficient to explain the reaction to art-making. Surprisingly, the largest suppression of PNS and augmentation of the SNS occurred during art-making with oil-pastels and not with Gouache. Moreover, PNS and SNS reactivity to oil-pastels were related to emotional valance, which may point to emotional engagement. We can conclude that art-making with oil-pastels, first created in Japan in 1924 to increase self-expression of students, results in a unique emotional and physiological responses. These findings might be explained by the enhanced tactile experience of art-making with oil-pastels along with their relative fluidity, triggering an arousal pattern. Further studies that take the format and presentation of the materials as well as the content of the artwork, into account, are needed.

## Introduction

Art therapy is a mental health profession that encourages the use of art materials to express feelings and thoughts as part of a supportive relationship. This process has been shown to promote improved mood (Drake et al., 2011), catharsis and stress reduction (Curl, 2008). An experimental study examined the effect of drawing with a black marker on white paper as compared to writing following a negative mood induction. The researchers found that drawing led to increased positive affect in comparison to writing. Furthermore, using art-making (and writing) for distraction rather than for venting, promoted positive mood (Drake et al., 2011). The opportunity to choose what to create as well as the art materials and art-making techniques with which to create, enhances one’s ability to solve problems, make decisions, and act upon them (Foster, 1992; Moon, 1994; Czamanski-Cohen, 2012).

The expressive therapies continuum (ETC) is a theoretical model for the assessment and application of media in art therapy (Lusebrink, 2010). The ETC can be utilized to assess the predominance of sensory information processing based on the configuration of formal elements in the art work and the interaction of the art-maker with the materials. The ETC also accounts for the effect of the texture and fluidity of the art materials, which are postulated to differentially effect the level of sensory information processing as well as the potential to elicit emotion (Kagin and Lusebrink, 1978; Lusebrink et al., 2013). For example, pencils and markers are highly structured, easy to control, and are assumed to promote limited emotional arousal. Oil-pastels are soft and easily smeared, thus their use entails tactile engagement and encourages emotional arousal (Hinz, 2009; Moon, 2010). Finally, gouache paint is an aqueous material with the potential for regressive engagement and high levels of emotional arousal. Gouache is used with a brush, which offers structure and some sense of control (Snir and Regev, 2013).

Several studies interviewed art therapists and art therapy clients about their experience of engaging in art therapy. Snir and Regev (2013) analyzed 30 reflective writing samples of students after working with five different art materials. They found that students reported a sense of pleasure in working with materials that entailed a risk of getting dirty and losing control. However, these materials also led to the fear of getting dirty and an attempt to balance flow and control. Their overall conclusion is that the art-making experience is related to the interaction between the material qualities, the art-maker’s personal attributes and previous art-making experiences. An experiment examined the effect of 41 children engaging in art-making with pencils, oil-pastels, and gouache paint over 10 group sessions. The researchers found decreases in aggression in the group that used gouache, however, not pencils or oil-pastels. Furthermore, there were no changes in self-esteem, anxiety, or self-control following the group art-making (Passo-Aviv et al., 2014). Two studies qualitatively investigated the opinions and experience of art therapists of the use of art materials in art therapy assessments (Pénzes et al., 2014, 2015). These authors used focus groups and interviews to create a categorization of the ways in which art therapy clients interact with art materials. They concluded that art therapists observe how clients interact with art materials to assess cognitive and emotional characteristics. In addition, the authors claim that these studies confirm the ETC claim that resistive art materials lead to cognitive processes while fluid materials promote affective processes.

However, this conclusion is based on art therapists’ clinical experiences rather than empirical knowledge. To support the theoretical base of the art therapy profession, empirical studies of an experimental nature along with clinical trials need to be conducted. The Bodymind model of Art Therapy (Czamanski-Cohen and Weihs, 2016) delineates several mechanisms through which Art Therapy benefits clients. The model is developmental and epigenetic in nature, meaning that each therapeutic process described develops from a preceding process in a cyclical manner (Wynne, 1988). The processes begin with the triangular relationship between the client- the art- therapist and the art-making process and product, which is elementary in any art therapy setting. It proceeds to self-engagement that is possible after a basic security in relationship is established. The self-engagement emphasizes the self as the integration of body and mind when engaged in art therapy. The third process is the embodied self-expression that occurs when emotional content transitions from implicit to explicit expression which can occur on a continuum in which content remains embedded in a visual metaphor to a verbal discussion during and following art-making. Lastly, meta-cognitive (thinking about thinking) processes include mechanisms that entail exploration and reflection of the self, the art-making process and product as well as relationships in and outside of the art therapy setting. Each core process is further divided in to several mechanisms that can be measured in experimental or clinical studies. The mechanisms can overlap and occur under several processes, or just one, depending on their nature. The core processes are cyclical rather than linear and can be revisited over sessions or within one session. The bodymind model has two main goals- to describe how art therapy may have a salutary effect on individuals and to provide a clear framework for the conduct of studies that examine how art therapy benefits clients. This study follows the latter call to empirically examine psychological and physiological responses following art-making. One proposed physiological measure with cognitive and emotional implications is heart rate variability (HRV).

The autonomic nervous system (ANS) is the primary neural mediator of physiological responses to internal and external stimuli and is comprised of the sympathetic nervous system (SNS) and parasympathetic nervous system (PNS). The two branches of the ANS usually act in a reciprocal opponent fashion on target organs. However, the balance between sympathetic and parasympathetic activity is not a simple bipolar unimodal process and under some physiological conditions, both branches are enhanced or inhibited at the same time (Teff, 2008). The SNS has an activating role and stimulates the body’s fight-or-flight response to stressful situations while the PNS has an inhibiting role and promotes relaxation (Porges and Byrne, 1992). The HRV index is a non-invasive way to gauge ANS activity (Porges and Byrne, 1992). Fluctuation in the intervals between heartbeats comprise the HRV index and express the responsiveness of the cardiovascular and nervous systems to the constantly changing external and mental environment (Cohen and Taylor, 2002).

The vagus nerve is the longest nerve in the ANS and it innervates many of the body’s organs. The vagus is also partially responsible for the ANS innervation of the heart. The myelinated vagus actively attenuates the SNS’s influences on the heart. The most influential theoretical frameworks that guide a large portion of HRV studies emphasize the neural influence that affect behavioral flexibility following changes in the physical, emotional, and social environment. Porges’s (2007 & 2011) Polyvagal theory emphasizes the importance of this system in response to emotional and social stimuli. The neurovisceral integration model (NIM, Thayer and Lane, 2000) also emphasizes the role of the vagus in adapting to the environment. The NIM focuses on HRV as indicative of vagal inhibition of the heart and reflecting the primary output of the ANS. The NIM also provides the platform for further studies that associate brain activity and functioning, that are beyond the scope of this paper. Both models are complementary and have differing, but not contrasting emphases on the role of vagus nerve, reflected in HRV. The NIM postulates that higher resting HRV indicate tendencies for appraisal while the Polyvagal theory associates higher levels of HRV with a capacity for social engagement (Kemp et al., 2017).

Heart rate variability at rest is indicative of ANS health. High variability is associated with a range of motion and flexibility in physiological processes, adaptive response, and good adaptability to changes. Therefore, people with low HRV at rest tend to be less flexible both physiologically and behaviorally in adapting to environmental demands (McCarty et al., 1995; Lane et al., 2009; Meerwijk et al., 2014). Low HRV at rest was found to be associated with difficulties in emotional regulation (Williams et al., 2015) and psychiatric conditions (Adrian et al., 2011). However, when participants used an effective strategy for emotional regulation (reassessment), the difference in resting HRV between anxious and non-anxious individuals was reduced (Reinecke et al., 2015).

Heart rate variability reactivity is a change from the basal state in response to stimuli. An increase in the respiratory sinus arrhythmia (RSA) index, which is a high frequency index of HRV, indicates an increase in heart’s vagal cardiac control, which is reflected in a decrease in heart rate (HR), and a decrease in the RSA indicates a withdrawal in the vagal effect on the heart, which is reflected in an increase in HR (Malliani et al., 1994; Malik, 1996; Porges, 2007; Rottenberg et al., 2007; Meerwijk et al., 2014). High volatility, theoretically attesting to high adaptability, is associated with high attention processing and regulatory capabilities (Suess et al., 1994). The emotional response to stimuli results in a decrease in RSA (Yaroslavsky et al., 2013).

Two other mechanism proposed by the Bodymind model (Czamanski-Cohen and Weihs, 2016) are tactile engagement and relaxed arousal, both measured using the self-assessment manikin visual analog scale (SAM; Bradley and Lang, 1994). Tactile engagement relates to the experience of interacting with the art materials that begins in the exploratory and pre-symbolic stages of art-making. This mechanism is postulated to lead to the experience of pleasure related from a sensory experience. The SAM can measure the intensity of that response using the dominance sub-scale. The tactile engagement with the art materials can lead to a state of arousal that is well-balanced with relaxation. The level of arousal and relaxation can be measured using the SAM. As well as by looking at changes in sympathetic and parasympathetic indices of HRV.

The objective of the current study was to examine the theoretical claim that art-making with art materials with different levels of fluidity (pencil, oil-pastel, and gouache paint) creates a differentiated emotional and thus physiological response. To obtain this objective, we conducted a controlled experiment that measured the mechanisms of emotional response and HRV during art-making with three art materials. Hypothesis 1: Drawing with different materials will differentially affect emotional response. We hypothesize that drawing in pencil will result in the lowest while painting in gouache will result in the highest arousal. Hypothesis 2: Art-making will affect HRV compared to baseline. Mean change in the parasympathetic function will be largest while drawing in pencil and smallest while painting in gouache. Conversely, we expect an increase in sympathetic function while engaged in art-making, with the smallest change expected during drawing with pencil, as it requires cognitive engagement and planning as compared to drawing with oil-pastels and gouache paint, respectively. Hypothesis 3: HRV and self-reported emotional response after art-making will be related. As emotional response increases we expect to see decreases in the parasympathetic index and increases in sympathetic index.

## Materials and Methods

### Research Procedure

The study was approved by the Ethics Committee of the Faculty of Health and Welfare at the University of Haifa. The participants were recruited using the snowball method through which the experiment was advertised and interested participants were requested to invite friends and acquaintances to participate in the study. Conditions while measuring HRV were maintained by having the subjects in the laboratory between 08:00 and 15:00 to ensure similar environmental conditions. Per guidelines for experimental studies measuring HRV (Thayer et al., 2008; Quintana et al., 2016), and to create baseline conditions, 2 h before the experiment subjects were asked to avoid drinking coffee, eating, smoking, and exercising. Two video cameras were installed in the room that was activated after signing the informed consent form and were filmed throughout the experiment. After attaching a wearable Electrocardiogram (ECG) device and adapting to the environment, 5 min of resting HR was measured in a sitting position to establish baseline HRV.

**FIGURE 1:**
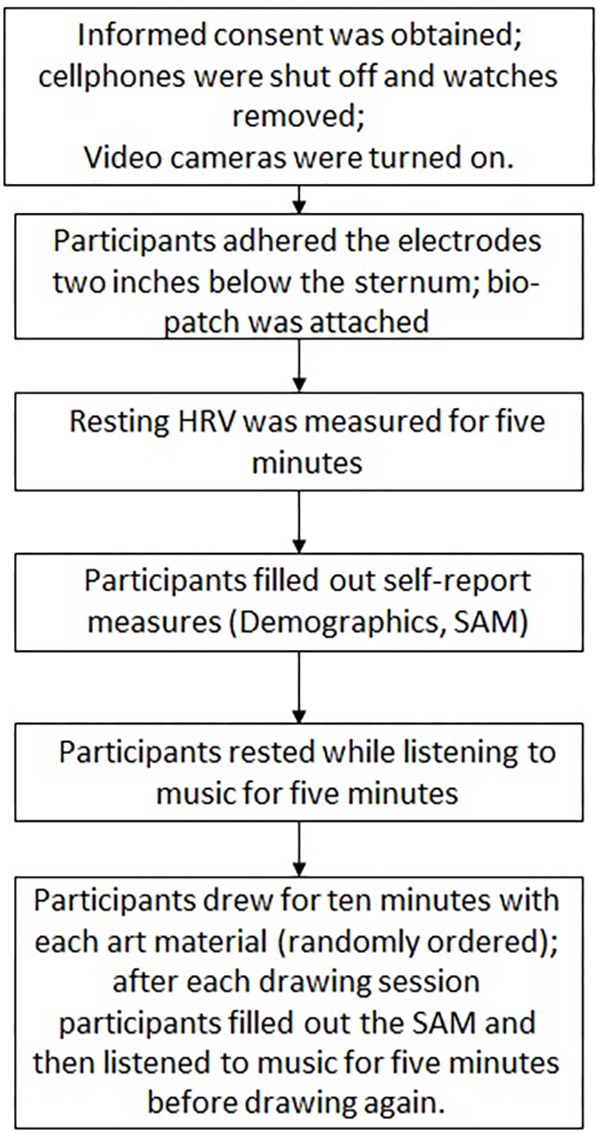
Flow chart of the experiment.

Each participant engaged in three 10 min art-making sessions (pencil, oil-pastels, and gouache paint) (see **Figure 1**). The order of the art materials was randomized to ensure that the effect of drawing with the material was not affected by the order of the presentation. To establish a baseline in terms of arousal, participants listened to 5 min of relaxing music of their choice (nature sounds, new age, or classical music) before each art-making session. The rationale for the music listening was to create a comparable condition before the art-making session and ensure equal conditions within participants. After each art-making session, participants reported their emotional response using a visual analog scale, described below. The subjects received a (50 cm × 35 cm) sheet of paper and were instructed to draw for 10 min. Participants were encouraged to use the entire 10 min for art-making. The pencil was provided with a pencil sharpener and eraser. An open box of 12 colors of oil-pastels was placed on the table, and participants were told that they can use them in any way they choose including peeling the wrapper and breaking the pastel if needed. The box of pastels was replaced for the next subject if their general appearance was significantly affected by use. Gouache paint in primary colors (yellow, red, and blue), black and white were presented in a plastic palate, divided into six bowls and four mixing surfaces, to control the quantities of paint. In addition, a color mixing chart, a jar of water, a soft, flat-headed brush (size 6) and a cloth were provided. The subjects were told that they could request additional paint if needed. The researcher provided a short explanation of mixing colors and cleaning the brushes.

### Participants

Sixty adults participated in the study (30 women). Inclusion criteria were: normative development and health, Hebrew speakers at a level sufficient for their participation between the ages of 18 and 60. Exclusion criteria were current or history of heart disease. The study included 60 subjects, 30 men and 30 women between the ages of 21 and 56 years, with an average age of 33 ± 10.27 years, 38 of them were born in Israel (63.3%), 18 in the former USSR (30%) and others in different countries. 28 of them were single (46.7%), 30 were married (50%), and 2 divorced (3.3%). In terms of education, 20 of them have a high school education (33.3%), 23 have a bachelor’s degree (38.3%), 18 have a higher degree (30%), and seven have a professional certificate (11.7%). Fifty-nine of the participants were Jewish (98.3%), and 51 defined themselves as secular (85%). Secular Jews, are individuals who are Jewish by ethnicity, but do not define themselves as religious (Cohen and Susser, 2000). 40 were not engaged in art (66.7%), 14 were engaged in art as a hobby (23.3%), and six professionally (10%). Four of them reported that they were in emotional therapy (6.7%) and 11 were taking medication regularly (18.3). There was no attrition during the study.

### Research Tools

#### Self-Report

The practice of art was coded by a single item “Do you regularly engage in art-making?” to which the participants marked one of three possible answers: “No” (1), “Yes, as a hobby” (2), “Yes, professionally” (3).

Emotional responses were measured with the self-assessment manikin visual analog scale (SAM, Bradley and Lang, 1994), which is a valid nine-point visual scale for measuring the valence, arousal, and dominance of emotional response. The valence scale ranges from unhappy or sad to happy or joyful. The arousal scale ranges from calm or bored to stimulated or excited. The dominance scale ranges from submissive or “sense of being without control” to dominant or “in control” (Lang, 1980). Following Tsonos and Kouroupetroglou (2008) SAM scales were centered on zero ranging from -4 to 4. Valence and arousal have been validated against physiological responses related to emotional experiences such as HR, skin conductivity and electrical activity of facial muscles (Bradley et al., 1992; Lang et al., 1993).

#### Heart Rate Variability (HRV)

The frequency of the HR is calculated using spectral analysis by calculating the difference in time between each beat [inter-beat interval (IBI)]: high frequency (HF: 0.15–0.4 Hz), low frequency (LF: 0.04–0.15 Hz), and very low frequency (VLF: 0–0.04 Hz) (Malliani et al., 1994). The high-frequency component of the HRV is associated with vagal activity that regulates respiration rate and represents RSA. Low RSA indicates increased activity of the PNS is indicated in a lower RSA index. We assessed sympathetic function can be using a non-linear method to analyze fluctuations in heartbeats using a Lorenz plot and resulting in two indices that point to sympathetic (CSI) and parasympathetic (CVI) function (Toichi et al., 1997; Allen et al., 2007), also coined Non-Linear SD1/SD2 (Kemp et al., 2017). These measures have been field validated and found to have factor structures and patterns of changes from baseline to stressor consistent with indices of parasympathetic and sympathetic activity, except CVI, which was not found to discriminate between baseline and stressor (Hibbert et al., 2012).

Heart rate variability was estimated based on continuous ECG recordings, at rest and while drawing. ECG was sampled (1000 Hz, 16 bit) and recorded by a small wireless and portable device (BioPatch, Zephyr Technology, Annapolis, MD, United States), that was attached to the participants’ chest using Ag–AgCl disposable electrodes. ECG series were visually inspected for artifacts since ECG artifacts might be quite similar to those of R waves, it may result in R–R, i.e., IBIs miscalculations. The R component of the ECG was identified using the QRS-tool software. ECG series was visually inspected alongside the identified heartbeats time points and artifacts (missed or wrongfully identified heartbeats) were corrected manually (Allen et al., 2007). The two indices of HRV (RSA- calculated by the extraction of frequencies of variability and CSI- using a Lorenz plot to analyze fluctuations in heartbeats) were calculated using CMetX software (Allen et al., 2007), is a command-line based utility that calculates various matrices of HRV given a simple IBI series as an input. HRV reactivity was calculated by subtracting the mean HRV index during music listening from the HRV index during the following art-making session (i.e., positive values represent higher HRV during art-making compared to the proceeding relaxation).

### Statistical Methods

To compare HRV in the different experimental conditions, a repeated measures ANOVA was performed. In the planned pairwise comparisons, *t*-tests were performed, whereas in unplanned comparisons a *post hoc* test of Sidák was used. To compare the experimental conditions with measurements of a rank scale, a non-parametric Friedman’s test was performed. For the pairwise comparisons, we conducted the Wilcoxon test. To test the correlation between variables, Pearson or Spearman correlation coefficients were calculated.

## Results

### Demographic Data

Fifty of our participants (83%) were under the age of 37 while 10 participants were between 40 and 60. Since the effect of age on RSA was determined, we decided to examine differences in HRV at baseline between the two age groups. Baseline RSA in participants over the age of 40 5.15 (± 0.70.) was found to be statistically significantly lower than RSA in subjects under the age of 40 6.32 (± 1.21) [*t*(58) = 3.15, *p* = 0.003, *Cohen’s d* = 1.184]. Because lower RSA due to age difference may mask the responsiveness to different materials of each group, we decided to omit them in our analyses. We also identified further differences in demographics between the two age groups (**Table 1**). We included 50 participants between the ages of 21 and 36 (see **Table 1** for full demographic data). No differences were found in HRV, indexed as RSA [*t*(48) = 0.77, *p* = 0.442, *Cohen’s d* = 0.213] between men [0.643 (± 1.08)] and women [6.19 (± 1.71)].

**Table 1:** Demographic data and comparison between age group.

Variable		*N*
Sex	Male	26
	Female	24
Birth country^∗∗^	Israel	37
	Other	13
Marital status^∗∗^	Married	20
	Other	30
Parenthood^∗∗∗^	Have children	11
Education^∗^	High school	10
	Certificate studies	6
	BA	23
	MA/Ph.D.	11
Employment	Work	41
	Not working	9
Religion	Jewish	49
	Christian	1
Religiosity	Secular	43
	Traditional	4
	Religious	2
	Other	1
Experience with art	No	32
	As a hobby	13
	Professionally	5

*^∗^p < 0.05; ^∗∗^p < 0.001; ^∗∗∗^p < 0.0001.*

It is plausible that participants who practice art on a regular basis may respond to the experiment differently than those who do not practice art. Therefore, we decided to examine differences between the average physiological indices in the baseline condition between individuals with habitual art-making experience and those without. No difference was found at baseline RSA [*t*(48) = -0.54, *p* = 0.052, *Cohen’s d* = -0.15] between those who practice or do not practice art.

To examine whether listening to relaxing music could be used as a baseline for each task we conducted an analysis of variance between the different time-points in which participants were listening to relaxing music as well as the baseline HRV measure at the beginning of the experiment. There were no statistically significant differences between the baseline levels (*M* = 6.32, *SD* = 0.16) and music listening (before pencil drawing *M* = 6.34, *SD* = 0.15, before oil-pastel painting *M* = 6.39, *SD* = 0.15, and music before drawing in gouache *M* = 6.35, *SD* = 0.17) [*F*(3,147) = 0.24, *p* = 0.868, $\eta_{p}^{2}$= 0.005]. Hence, we conclude that the state of listening to the music before each material can be considered a baseline measurement.

### Emotional Response and Art-Making (H1)

The first hypothesis was that painting with different materials would result in an emotional response in which pencil would have the least effect, while gouache would have the largest effect. In the analysis of the variability of the emotional valance, a significant difference was found between the experimental conditions [*F*(3,147) = 2.93, *p* = 0.030, $\eta_{p}^{2}$= 0.056]. In a Sidák’s *post hoc* test to examine the source of the difference, we found that increased positive valance was found following painting with gouache in comparison to emotional valence following drawing with a pencil (*p* = 0.038), but not in comparison to baseline valence (*p* = 0.744). Contrary to our hypothesis, there was no difference in emotional arousal nor dominance following art-making with the different art materials (**Table 2**).

**Table 2:** Valence, arousal, and dominance in baseline and art-making with different materials (mean, SD, and variance analysis).

	Baseline	Pencil	Oil-pastel	Gouache	*F*^(3,147)^
	
	(M/SD)	(M/SD)	(M/SD)	(M/SD)	(M/SD)
Valence	2.14 (± 1.07)	1.96 (± 1.65)	2.30 (± 1.54)	2.60 (± 1.55)	2.93^∗^
Arousal	-1.72 (± 1.84)	-1.86 (± 2.13)	-1.90 (± 1.88)	1.66 (± 2.11)	0.47
Dominance	-0.02 (± 1.41)	0.16 (± 1.54)	0.14 (± 1.77)	0.16 (± 1.82)	0.28

*^∗^p < 0.05.*

### The Relationship Between Art-Making and HRV (H2)

The second hypothesis was that mean RSA would decrease and CSI would increase from baseline in each material. This hypothesis was confirmed in RSA, but not in CSI. The results of the analysis indicated an interaction between material and state, with a decrease in mean RSA from resting state while painting [*F*(1,49) = 26.155, *p* < 0.0005]. The effect size ($\eta_{p}^{2}$= 0.348); indicates that 35% of the variability of RSA is explained by art-making, regardless of art material. When examining the differences in RSA for each of the materials, the smallest change was as expected during art-making with pencil 6.22 (± 01.020) compared to baseline 6.34 (± 01.008) [*t*(49) = 1.40, *p* = 0.168, *Cohen’s d* = 0.105]. But contrary to the hypothesis, the greatest change was found to be during art-making with oil-pastels 5.90 (± 0.99) compared to baseline 6.39 (± 1.07) [*t*(49) = 5.51, *p* < 0.0005, *Cohen’s d* = 0.475] and not during art-making with gouache 6.01 (± 1.09) compared to baseline 6.35 (± 1.21) [*t*(49) = 3.63, *p* = 0.001, *Cohen’s d* = 0.195].

In examining the change in RSA from baseline (ΔRSA), a statistically significant difference was found between the experimental conditions [*F*(2,98) = 5.97, *p* = 0.004]. The effect size ($\eta_{p}^{2}$= 0.109) indicates the variance of ΔRSA explained by the drawing material. The change while drawing with a pencil was small in comparison to oil-pastel (*p* = 0.007), but not gouache (*p* = 0.099). There was no difference in the change in RSA between drawing with oil-pastel and painting with gouache (*p* = 0.412). The change in CSI from baseline (ΔCSI), was significantly different in the experimental conditions [*F*(2,58) = 3.98, *p* = 0.024] with an effect size ($\eta_{p}^{2}$= 0.24) indicating that the variance of ΔCSI is explained by the drawing material. The change in CSI after drawing with a pencil is smaller than the change in CSI following drawing with oil-pastels (*p* = 0.025). The difference in changes between CSI during art-making with oil-pastels compared to painting with gouache was also significant (*p* = 0.02). There was no difference in the changes in CSI following painting with gouache and drawing with pencil (*p* = 1.0). The means, standard deviations, and results of analyses of this index in the state of the various materials appear in **Table 3**.

**Table 3:** RSA, Δ RSA CSI, and ΔCSI during art-making with different materials (mean, SD, and variance analysis).

	Pencil	Oil-pastel	Gouache	*F*^(2,98)^
		
	(M/SD)	(M/SD)	(M/SD)	
RSA	6.23 (± 1.07)	5.90 (± 0.98)	6.01 (± 1.09)	3.58^∗^
ΔRSA	-0.11 (± 0.57)	-0.49 (± 0.62)	-0.33 (± 0.65)	5.97^∗∗^
CSI	2.85 (± 0.14)	3.04 (± 0.13)	2.83 (± 0.13)	0.017
ΔCSI	-0.29 (± 1.02)	0.08 (± 1.06)	-0.15 (± 0.85)	7.41^∗∗^

*^∗^p < 0.01; ^∗∗^p < 0.001.*

### Examining the Association Between Emotional Response and HRV (H3)

The third hypothesis was that emotional response and HRV would be related (increased emotional response and increased changes in RSA and CSI). Contrary to hypothesis, we found no linear other relationship between emotional state (valence, arousal, and control) and HRV parameters (RSA/ΔRSA and CSI/ΔCSI).

## Discussion

The current study examined the effect of drawing with different art materials on HRV and emotional response. Leaning on the ETC model (Kagin and Lusebrink, 1978) and the Bodymind model of art therapy (Czamanski-Cohen and Weihs, 2016), we hypothesized that similar to the stages of visual information processing in the brain, art-making with fluid art materials would result in an increase in emotional response (Lusebrink et al., 2013). This research is unique in its examination of the relationship between the use of different art materials and HRV. It responds to the claim, presented in the Bodymind model, that places importance on conducting studies that examine the ways in which art-making in the framework of a supportive relationship has a salutary effect.

The hypothesis that creating with different materials would have a differential emotional response (pencil having the least and gouache the most) was partially confirmed by increase of emotional valance but not emotional arousal or control. Art-making, has been shown to increase positive mood (Drake et al., 2011) however we expected to find increases in arousal and intensity of emotion following engagement with more fluid art materials. According to Kapitan (2013), a certain amount of stress always accompanies the creative process and can be a driving force in the process. However, the ability to maintain equilibrium, between arousal and relaxation by require the increased ability for emotion and sensory regulation. The participants in this study were a normative population, most likely with effective adaptive and coping mechanisms, which may moderate their emotional response following art-making with the different art materials.

Flooding and withdrawal during the creative process can be motivated by self-judgment, performance anxiety, worry, stress, lack of attention and physical exhaustion (Kapitan, 2013). In this study, drawing with pencils resulted in the lowest increases in positive mood. Pencils are monochromatic and may create connotations to elementary school and be associated with cognitive (dis)abilities (McManus et al., 2010). This may give rise to self-criticism, thus making the art-making experience less pleasant. In addition, the paper presented to the subjects was the same for all the materials. The properties (size, thickness, and texture) of the drawing/painting platform influence its ability to contain aqueous materials (Moon, 2010). We chose a relatively large and thick sheet to contain the gouache paint which may have evoked unease during drawing with pencils.

Unlike pencil, gouache paint was associated with the highest increase in positive valence, similar to the aggression reduction found by Snir and Regev (2013). Memories of childhood are important in working with materials (Snir and Regev, 2013), and oil-pastels may be perceived as childish and less “artistic,” while painting by brush and palette, with which artists paint with oil colors and acrylic, may have been perceived as more mature and “professional.”

Art-making explained approximately 35% of the variability in parasympathetic reactivity, which may indicate changes in emotional regulation processes during the art-making task. However, fluidity alone was not sufficient to explain the emotional response to art-making. The hypothesis that art-making with different materials would affect RSA was confirmed to a large extent however, there was no significant difference in HRV after painting with gouache compared to after drawing with oil-pastels. We believe that this may be because of the mediation of the paintbrush as opposed to drawing with oil-pastels without mediation. The brush prevents direct contact with the paint which in turn limits tactile engagement (Kagin and Lusebrink, 1978; Czamanski-Cohen and Weihs, 2016). Therefore, even though gouache is more liquid than oil-pastels, oil-pastels may be experienced as “dirtier” because they smear and leave marks on the art-maker’s hands. In other words, it is possible that the intensity of the reaction to the painting material depends not only on the degree of liquidity of the material but on the degree of its contact with the art-maker’s skin. Another possible explanation is related to the relatively thin brush and the way the gouache colors were arranged on the palette in a limited quantity which may have increased the art makers’ sense of control. Possibly a thicker brush and more paint would enhance tactile engagement and result in greater changes in HRV during gouache painting. Surprisingly, the largest suppression of the PNS and augmentation of the SNS occurred during art-making with oil-pastels and not with Gouache. Moreover, PNS and SNS reactivity to oil-pastels were related to emotional valance, which may point to increased emotional engagement.

Drawing with oil-pastels demonstrated a differential pattern of sympathetic response in comparison to while drawing with pencil and painting with gouache paint. CSI decreased while working with both gouache and pencil while it increased while drawing with oil-pastels. This finding may also be indicative of emotional and cognitive efforts while using oil-pastels.

The context in which oil-pastels were invented as a media to encourage self-expression is interesting. Influenced by British and American art education, the Japanese educational system during the Taisho period the Jiyu-ga movement theorizing that free form drawing increases creativity (as opposed to teaching art through copying, which was the norm at the time) (Okazaki, 1984). In 1924 by teachers Rinzo Satake and Shuku Sasaki with the consult of Kanae Yamamoto, the artist who formed the Jiyu-ga movement, sought to create a new and local art material to increase self-expression in students (Ellis and Yeh, 1998). Yamamoto recommended that the new art material have vivid colors and a soft texture to enhance creativity (Sakura Color Products of America, 2018). As far as we know, these hypotheses have not been examined by the Japanese educational system, or elsewhere. Our study, can provide some support that oil-pastels are doing what they were designed to do. Further research is needed to examine the extent of the self- expression involved, beyond emotion and physiological response.

Our findings are also interesting considering the clinical preference of oil-pastels by art therapists for their tendency to promote self-expression while maintaining the art-maker with a sense of control (Snir and Regev, 2013).

Thirdly, we expected to find a differential connection between emotional response and HRV in response to the various art-making materials. The greater the hypothesized emotional response, the greater the increase in mean RSA and vice versa. However, no relationship was found possibly due to our sample of a healthy and normative individuals. These associations were found in a study of individuals coping with psychiatric disorders, such as depression (Meerwijk et al., 2014). Another possibility is that the lack of connection stems from the nature of the task. The purpose of this study was not to focus on a certain emotion, and subjects were instructed to draw freely. A relationship between emotion and RSA was found in a normative population when a manipulation was performed to stimulate certain emotions (Lane et al., 2009).

### Research Limitations

The benefits of this study should be considered along with its limitations. We had a relatively small number of subjects; therefore, it was not possible to examine differences in emotional response in different age groups. The small sample size may also explain why we did not find the expected differences in HRV between men and women which were found in larger sample (*N* = 1970) or when stress induction was used (Nugent et al., 2011). Even though drawing with different materials was expected to affect the ANS, the intervention in the current study did not include stress induction. Furthermore, while the non-linear calculating CSI, has been used in studies to measure the HRV index of sympathetic function (Toichi et al., 1997; Allen et al., 2007) and more recently validated in the field (Hibbert et al., 2012), other studies question whether HRV indices accurately reflect cardiac sympathetic regulation in short term measurement (Reyes del Paso et al., 2013; Heathers, 2014).

Another limitation lies in the way art materials were presented to the subjects. For example, in the case of the gouache paint, the brush served as an intermediary and prevented direct contact with the material. Furthermore, placing small amounts of paint on the palette may have limited tactile engagement and moderated its impact on emotional involvement and changes in HRV.

In addition, in our sampling participants were recruited using the snowball method, which can lead to homogeneity in demographics and increased motivation to cooperate with the researchers. 98% of our participants were Jewish, 86% were secular, and 68% had an academic education. In addition, the self-report questionnaires may reflect the respondent’s desire to please the researchers (Cook and Campbell, 1979).

### Recommendations for Further Studies

Due to the paucity of research on materials and art therapy, and the limitations of the research mentioned above, additional research is needed. In this study, we found the largest decrease in HRV during drawing with oil-pastels. This finding raises questions about the properties of the materials examined, and it would be interesting to examine the effect of additional materials that differ from each other by tactile engagement or fluidity.

In addition, art therapists work with a variety of populations and age groups. Considering the homogeneity of the sample (Jewish population, secular, working, and educated), and considering differences in HRV found in other studies in different age groups, it would be important to examine a wider range of populations and age groups. It would be interesting to compare the response to different art materials in different groups with the goal of designing appropriately tailored interventions.

## Conclusion

The importance of this research to the field of art therapy stems from the growing demand for practice from an evidence-based research standpoint. This study is based on the ETC model, which emphasizes the importance of interaction with creative materials. The results reinforce this model and support the claim that different art materials have a different effect on emotional response and physiology. This research clarifies the importance of art therapists making well-informed choices of art materials. We call for follow-up studies to continue examining the effect of art-making with different art materials and their association with emotion, cognition, and physiology.

## Author Contributions

JC-C: formulated the idea for this experiment with assistance and input from GG and SH-I; trained SH-I to collect, clean, and analyze HRV data; conducted the data analysis and writing the manuscript. GG: assisted in planning the experiment; trained SH-I in collecting HRV data as well as cleaning, and analyzing the HRV data; conducted the data analyses and formulated the data analysis plan, participated in writing the manuscript. SH-I: collected the data; conducted the data analysis with guidance and participated in writing the manuscript. This manuscript is derived from a thesis she wrote in as part of her MA studies at the University of Haifa School of Creative Arts Therapies.

## Conflict of Interest Statement

The authors declare that the research was conducted in the absence of any commercial or financial relationships that could be construed as a potential conflict of interest. The handling Editor and reviewer GK declared their involvement as co-editors in the Research Topic.
